# Integrative Multi-Omics Mendelian Randomization Reveals Oxidative Stress Mechanisms in Major Depressive Disorder, Bipolar Disorder, and Schizophrenia

**DOI:** 10.3390/antiox15020233

**Published:** 2026-02-10

**Authors:** Nanxi Li, Juan Wang, Sihao Chen, Tao Li

**Affiliations:** 1 Affiliated Mental Health Center & Hangzhou Seventh People’s Hospital and School of Brain Science and Brain Medicine, Zhejiang University School of Medicine, Hangzhou 310058, China; 2 Liangzhu Laboratory, MOE Frontier Science Center for Brain Science and Brain-Machine Integration, State Key Laboratory of Brain-Machine Intelligence, Zhejiang University, 1369 West Wenyi Road, Hangzhou 311121, China; 3 NHC and CAMS Key Laboratory of Medical Neurobiology, Zhejiang University, Hangzhou 310058, China

**Keywords:** oxidative stress, major depressive disorder, bipolar disorder, schizophrenia, integrative omics

## Abstract

Background: Oxidative stress (OS) has been widely implicated in pathophysiology of major psychiatric disorder. However, establishing robust causal links and delineating the specific molecular mechanisms involved continue to pose significant research challenges. Methods: We performed a multi-omics analysis focusing on 817 oxidative stress-related genes (OSGs) in major depressive disorder (MDD), bipolar disorder (BD), and schizophrenia (SCZ). We applied summary data-based Mendelian randomization (SMR), integrating large-scale genome-wide association studies for MDD, BD, and SCZ with quantitative trait loci datasets from both blood and brain tissues, including measures of DNA methylation, gene expression, and protein abundance. Results: Multi-omics integration yielded supportive evidence across blood and brain tissues implicating *ACE* and *ACADVL* in SCZ, where genetically predicted increases in their methylation, expression, and protein abundance were associated with reduced disease risk. *IGF1R* was associated with bipolar disorder (BD) risk in blood-specific analyses. Brain-specific analyses further nominated *ENDOG* as a candidate gene for SCZ. Single-cell SMR indicated that increased *ENDOG* expression was associated with higher SCZ risk in astrocytes, CD4^+^ naïve T cells, CD8^+^ effector T cells, and natural killer cells, suggesting a potential immune–brain interaction. Conclusions: This study provides multi-level genetic evidence supportive of a potential causal role for specific OSGs in major psychiatric disorders. We identify *ACE*, *ACADVL*, *IGF1R*, and *ENDOG* as candidate genes for further investigation, offering insights into epigenetic and transcriptional mechanisms that could inform future research on therapeutic targets.

## 1. Introduction

Psychiatric disorders such as major depressive disorder (MDD), bipolar disorder (BD), and schizophrenia (SCZ) represent major public health challenges worldwide, exerting profound social, functional, and economic burdens on affected individuals and society [1]. Over the past decades, numerous studies have identified several molecular and pathophysiological mechanisms underlying these disorders, including multiple genetic risk loci linked to neurodevelopment and synaptic plasticity pathways [2,3], epigenetics modifications [4], and dysregulation of neurotransmitter systems such as dopaminergic, serotoninergic, and glutamatergic signaling. In addition, accumulating evidence implicates neuroinflammation and the immune-related processes in their pathogenesis [5]. Despite these advances, the etiology of psychiatric disorders remains incompletely understood. A significant proportion of patients, particularly those with difficult-to-treat phenotypes such as treatment-resistant depression (TRD) [6], experience limited benefits from existing therapies, highlighting a critical unmet clinical need for integrative, mechanistic approaches that can unify these diverse molecular pathways, thereby informing the development of novel therapeutic and preventive strategies.

Oxidative stress (OS) occurs when excessive production of reactive oxygen species (ROS) overwhelms endogenous antioxidant defenses systems, leading to oxidative damage of DNA, RNA, and proteins [7]. The growing body of clinical studies implicate OS in the pathophysiology of major psychiatric disorders. For instance, individuals with SCZ exhibit heightened OS responses during the Trier Social Stress Test compared with healthy controls (HCs) [8], while patients with BD and MDD exhibit abnormalities in antioxidant defense mechanisms [9]. Pre-clinical studies further support a mechanistic role for OS, showing that peroxisomal dysfunction disrupts ROS homeostasis and promotes mitochondrial impairment, neuroinflammation, and ferroptosis, processes all implicated in psychiatric pathophysiology [10,11]. However, due to the influence of confounding factors and the potential for reverse causation, it remains challenging to establish a causal and biologically grounded connection between OS and psychiatric disorders.

In recent years, the growing availability of large-scale genome-wide association studies (GWASs) and diverse quantitative trait loci (QTL) datasets has enabled the application of advanced genetic causal inference methods, such as mendelian randomization (MR) and its extension, summary data-based MR (SMR). These approaches facilitate the identification of causal association between molecular exposure and complex traits, reveal regulatory mechanisms, and support the discovery of therapeutic targets. To date, only a limited number of studies have used MR to demonstrate causal links between OS-related biomarkers or antioxidant targets and psychiatric disorders [12,13]. However, few SMR-based investigations have systematically explored the causal roles and molecular mechanisms of OS in major psychiatric disorders through multi-omics integration, combining DNA methylation QTLs (mQTLs), gene expression QTLs (eQTLs), and protein QTLs (pQTLs). Moreover, as gene expression regulation is increasingly recognized to occur in a cell-type-specific manner, incorporating single-cell eQTLs datasets could yield deeper insights into whether OS-related gene expression in specific neuronal or immune cell populations contributes to disease susceptibility. Compared with analyses relying solely on bulk-tissue expression data, such cell-resolved, multi-omics approaches have been shown to significantly improve the translational success of drug discovery efforts [14].

In this study, we integrated data from the largest available GWASs with multi-omics QTL resources derived from blood and brain tissue, as well as single-cell eQTLs datasets from both brain and peripheral immune cells. Outcome datasets included GWAS summary statistics for MDD, BD, and SCZ. We focused specifically on OS-related genes (OSGs) and their associated molecular traits, including gene expression, DNA methylation, and protein abundance. Using a comprehensive analysis pipeline that combined SMR with colocalization analyses, we aimed to elucidate the regulatory mechanisms through which OSGs contribute to the pathogenesis of major psychiatric disorders and to identify potentially druggable targets for future therapeutic development.

## 2. Materials and Methods

### 2.1. Study Design

**Figure 1:**
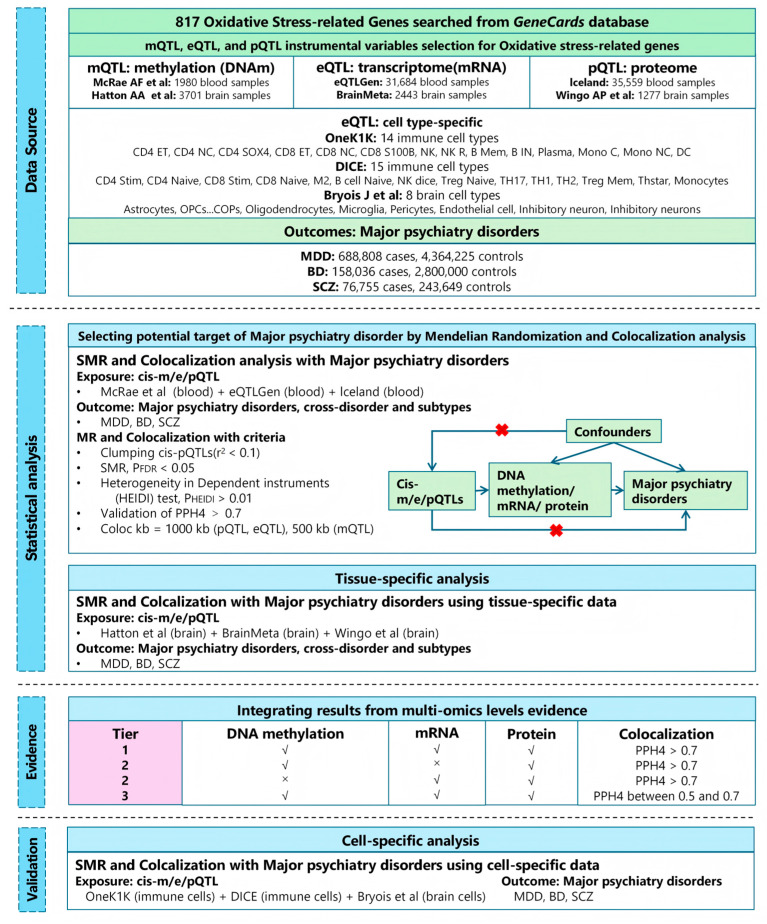
The research design and framework of this study. The diagram illustrates the stepwise analytical pipeline employed in this study. We first integrated large-scale genome-wide association study (GWAS) summary statistics for psychiatric disorders with oxidative stress-related multi-omics quantitative trait loci (QTL) datasets. These included DNA methylation QTLs (mQTLs), gene expression QTLs (eQTLs), and protein abundance QTLs (pQTLs). Causal inference was performed using the summary-data-based Mendelian randomization (SMR) method, complemented by Bayesian colocalization and the HEIDI (heterogeneity in dependent instruments) test to validate robustness and distinguish shared causal variants from linkage effects. The workflow concludes with cell-type-specific resolution using single-cell data.

Figure 1 illustrates the overall design of this study. We extracted summary statistics for mQTL, eQTL, and pQTL corresponding to OSGs from blood and brain datasets, and integrated them with GWAS summary statistics for MDD, BD and SCZ. Using an SMR framework, we investigate how methylation, expression, and protein abundance of OSGs may causally influence the risk of psychiatric disorders. To strengthen causal inference, we performed Bayesian colocalization analyses to distinguish shared causal variants from linkage effects. Candidate causal OSGs were then prioritized by integrating SMR results across molecular layers, followed by single-cell type expression analysis to explore whether the identified genetic effects were cell-type-specific. The study adhered to the STROBE-MR guidelines [15]. All datasets were publicly available, with ethical approval obtained in the original studies; therefore, no additional ethical approval was required for the present analysis.

### 2.2. Data Resources

#### 2.2.1. Multi-Omics Data of OSGs from Blood and Brain Tissues

A total of 817 OSGs were curated from GeneCards (relevance score ≥ 7) using the keyword “oxidative stress” (Supplementary File: Table S1). Blood eQTL summary statistics were derived from the eQTLGen consortium, comprising data from 31,684 individuals across 37 datasets [16]. Blood mQTL summary statistics were derived from 1980 individuals of European ancestry [17], and blood pQTL data were sourced from a study of 35,559 Icelanders [18]. To validate whether genetic effects detected in blood were also present in the brain, we extracted corresponding brain eQTL (*n* = 2443), mQTL (*n* = 411), and pQTL (*n* = 1277) datasets from previously published resources [19,20,21]. The analysis was restricted to cis-eQTLs, cis-mQTLs, and cis-pQTLs, which were defined as single nucleotide polymorphisms (SNPs) located within 1 Mb upstream or downstream of the target gene boundaries. Comprehensive information on all datasets used in this study is summarized in Supplementary File: Table S2.

#### 2.2.2. GWAS Summary Statistics for Psychiatric Disorder

As illustrated in Supplementary File: Table S2, GWAS summary statistics for MDD (688,808 cases; 4,364,225 controls) were obtained from the Psychiatric Genomics Consortium [22]. GWAS data for BD (158,036 cases; 2,800,000 controls) and SCZ (76,755 cases; 243,649 controls) were sourced from O’Connell et al. and Trubetskoy V et al., respectively [23,24].

#### 2.2.3. Immune and Brain Cell-Type-Specific eQTL Data

To further evaluate the cell-type specificity of genetically predicted effects identified through SMR analysis based on blood and brain tissues, we incorporated the immune- and brain cell-specific eQTL datasets from well-characterized European-ancestry cohorts. For the immune cell analyses, data were obtained from two large resources: (i) the OneK1K cohort, which includes single-cell RNA sequencing (scRNA-seq)-derived expression data of peripheral blood mononuclear cells (PBMCs) for 180 genes across 14 immune cell types from 982 donors (https://onek1k.org/); and (ii) the DICE consortium, providing eQTL data for 149 genes expressed across 15 distinct immune cell types (https://dice-database.org/). For the brain, we utilized cell-type-specific eQTL datasets encompassing eight brain cell-specific eQTLs (astrocytes, endothelial cells, excitatory neurons, inhibitory neurons, microglia, oligodendrocytes, oligodendrocyte precursor cells, and pericytes), primarily derived from the prefrontal and temporal cortices of 192 European individuals [25]. These data were used to perform single-cell Mendelian randomization analyses aimed at delineating cell-type-specific genetic effects across neural and immune contexts.

### 2.3. Statistical Analysis

#### 2.3.1. SMR and Colocalization Analysis Based on Data from Blood and Brain Tissues

OS-related genes were extracted from the GeneCards database (v5.10, https://www.genecards.org) using the keyword “oxidative stress” with a relevance score > 7. It is important to note that this approach yields a curated, hypothesis-driven gene set based on current annotation evidence, and is not intended to be an exhaustive list of all OS-related genes. We then performed SMR analysis to estimate the genetic associations between OSG-related molecular traits and the risk of major psychiatric disorders using the SMR software (v1.3.1). This approach leverages top associated cis-QTLs as instrumental variables, offering higher statistical power than conventional two-sample MR when exposure and outcome data are derived from large, independent samples [26].

Prior to analysis, all summary statistics (QTL and GWAS) were aligned to the GRCh37/hg19 genome build. We implemented a strict allele harmonization pipeline: SNPs were matched by rsID and chromosomal position; all alleles were aligned to the forward strand. Palindromic SNPs (A/T, C/G) with intermediate allele frequencies (0.42 < MAF < 0.58) were excluded to prevent strand ambiguity. SNPs exhibiting an allele frequency difference greater than 0.2 between any pairwise combination of the LD reference panel, QTL data, and GWAS data were also excluded. Linkage disequilibrium (LD) calculations were based on the 1000 Genomes Project Phase 3 European reference panel.

For each trait, the top associated QTLs were selected based on the following: (1) the *p* < 5 × 10^−8^ was applied to select significant SNPs from the QTL summary data to define genetic instruments; (2) the SMR analysis was performed using these instruments against the complete GWAS summary statistics. The analysis window was defined as ±1000 kb around the target gene for eQTL and pQTL, and ±500 kb for mQTL. The SMR test was then conducted using these instruments against the full GWAS summary statistics (without pre-filtering the GWAS data). The HEIDI test was applied to distinguish pleiotropy from linkage (i.e., to assess whether the SMR signal was driven by a single causal variant). A threshold of *p* < 0.01 to detect (and thus *p* > 0.01 to retain) has been employed in previous study [1]. This choice reflects a balance between the risks of Type I (false positive) and Type II (false negative) errors. While a more stringent threshold (e.g., *p* > 0.05) would more aggressively remove SNPs with potential pleiotropy, it may also over-exclude valid instrumental variables. Therefore, a HEIDI test *p*-value > 0.01 was interpreted as a lack of strong evidence against the pleiotropy model (i.e., supporting a causal link), while a more stringent threshold of *p* > 0.05 was used in sensitivity analyses to assess robustness. To account for multiple testing across numerous gene–trait associations, we applied the Benjamini–Hochberg procedure to control the false discovery rate (FDR) at α = 0.05 within each molecular layer (mQTL, eQTL, and pQTL analyses separately). A putative causal association was considered significant if it met both an FDR-adjusted pSMR < 0.05 and pHEIDI > 0.01.

We performed Bayesian colocalization analysis using the coloc R package (v5.2.3) to evaluate whether the QTL and GWAS association signals shared a common causal variant [27]. The default prior probabilities were employed as follows: p1 = 1 × 10^−4^ (prior for a SNP associated with the molecular trait), p2 = 1 × 10^−4^ (prior for a SNP associated with the psychiatric disorders), and p12 = 1 × 10^−5^ (prior for a SNP associated with both traits). The analysis tests five mutually exclusive hypotheses (H0-H4), with a posterior probability for H4 (PPH4) > 0.70 considered strong evidence for colocalization, corresponding to a FDR of <0.05 [28].

#### 2.3.2. Integrating Evidence from Multi-Omics Levels

To systematically investigate the association between OSGs and psychiatric disorders, we integrated evidence across three molecular regulatory layers: DNA methylation, gene expression, and protein abundance. Since proteins represent the functional endpoints of gene activity [29,30], we required all candidate genes to show a significant association at the protein level as the foundational criterion. Using a structured, tiered system, we prioritized genes by cross-validating significant signals identified in independent SMR analyses [31]. Candidate genes were categorized into three evidence tiers according to the consistency of associations across the molecular cascade, based on the following criteria: (1) Tier 1: Genes showing significant associations with psychiatric disorders at the protein abundance level (FDR-corrected *p* < 0.05 and PPH4 > 0.7), and significant associations at both the methylation and expression levels (FDR-corrected *p* < 0.05). (2) Tier 2: Genes showing significant associations with psychiatric disorders at the protein abundance level (FDR-corrected *p* < 0.05 and PPH4 > 0.7), and associations at either the methylation or expression levels (FDR-corrected *p* < 0.05). (3) Tier 3: Genes showing significant associations with psychiatric disorders at the protein abundance level (FDR-corrected *p* < 0.05 and 0.5 < PPH4 < 0.7), and associations with psychiatric disorders at both methylation or expression levels (nominal *p* < 0.05). This process constituted not a further statistical analysis, but a structured, consensus-based prioritization framework aimed at identifying robust, multi-layer signals.

#### 2.3.3. Further SMR Analysis Based on Single-Cell eQTL Data

After integrating candidate OSGs associated with psychiatric disorders at the tissue level, we conducted additional SMR to investigate cellular-level associations between OS and psychiatric phenotypes. To maintain methodological consistency, we employed the same stringent criteria for selecting instrumental variables derived from immune cell-specific and brain cell-specific eQTLs datasets corresponding to the identified causal genes. These criteria included LD clumping (*r*^2^ < 0.001), an F-statistic > 10, and the application of Steiger filtering to confirm the directionality of causal effects.

## 3. Results

### 3.1. Blood OSGs Methylation and Major Psychiatric Disorders

**Figure 2:**
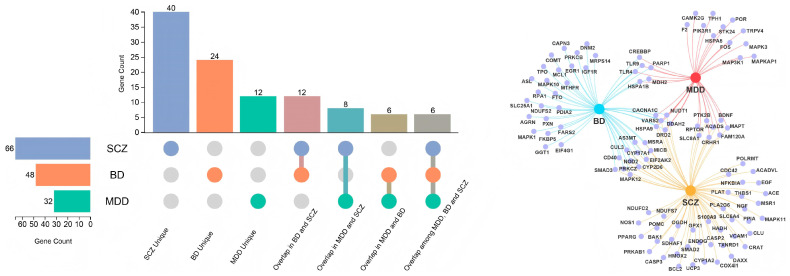
Assessing the relationship between oxidative stress-related DNA methylation sites corresponding to genes and psychiatric disorders. Left: marginal-bubble visualization of the relationship between oxidative stress-related DNA methylation sites corresponding to genes and psychiatric disorders. Right: network Venn diagram of shared and distinct genes among psychiatric disorders driven by oxidative stress-related differentially methylated DNA sites corresponding to genes. MDD, major depressive disorder; BD, bipolar disorder; SCZ, schizophrenia.

The results of SMR analyses assessing associations between DNA methylation of OSGs and the three psychiatric disorders are summarized in Figure 2 and Supplementary File: Table S3. After multiple testing correction, we identified 79 significant CpG loci near 32 OSGs associated with MDD, among which 23 loci spanning 10 genes (*CREBBP*, *CRHR1*, *FAM120A*, *MAPK3*, *MAPT*, *MDH2, POR*, *SLC8A1*, *STK24*, and *VARS2*) showed colocalization evidence (PPH4 > 0.70). For SCZ, 158 significant CpG loci near 66 OSGs were identified, with 46 loci across 20 OSGs supported by colocalization evidence. Similarly, for BD, 129 significant CpG loci near 48 OSGs were identified, with 50 loci across 16 OSGs showing evidence of colocalization. Notably, several genes exhibited methylation-site-specific effects on disease risk. For example, in *ACADVL*, a one standard deviation (SD) increase in methylation at cg16564940 was associated with a reduced risk of SCZ (OR = 0.865, 95% CI: 0.787–0.951, PPH4 = 0.79), whereas increased methylation at cg13768953 was associated with an elevated risk of SCZ (OR = 1.054, 95% CI: 1.025–1.084, PPH4 = 0.65) (Figure 2).

### 3.2. Blood OSGs Expression and Major Psychiatric Disorders

**Figure 3:**
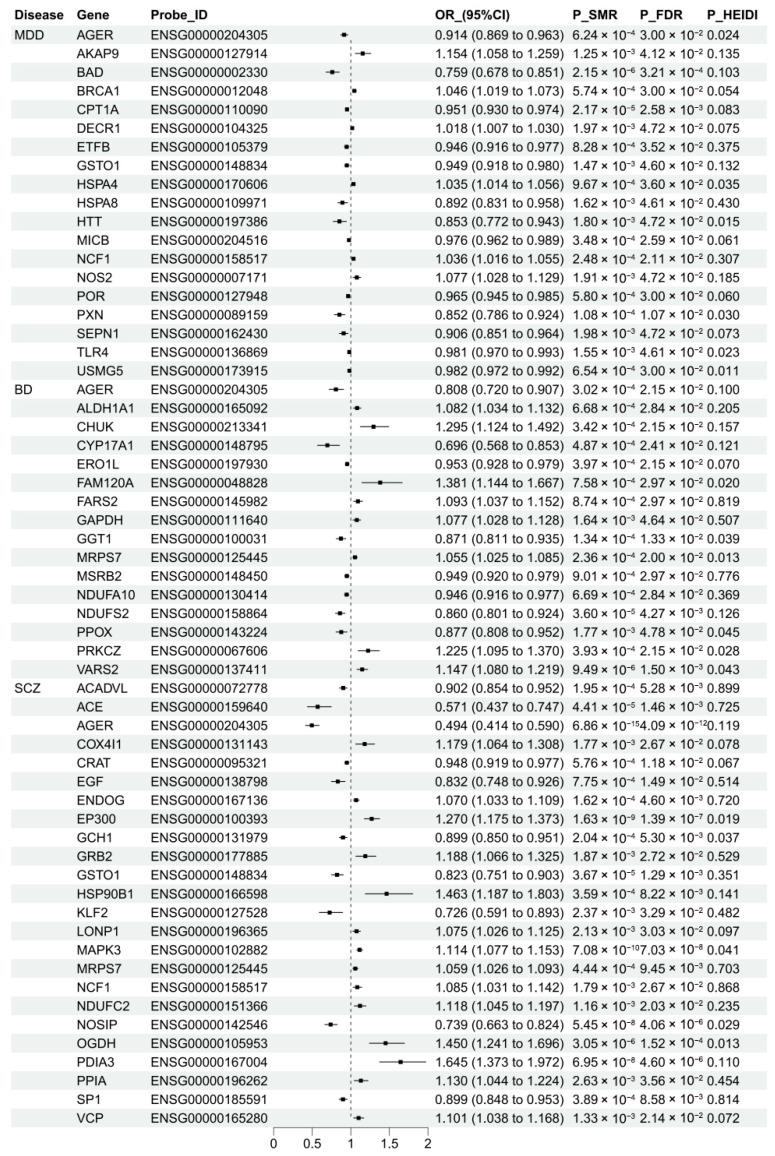
Assessing the relationship between oxidative stress-related gene expression and psychiatric disorders. The figure presents findings from summary-data-based Mendelian randomization (SMR) analysis, showing associations that met the threshold of false discovery rate (FDR)-adjusted *p* < 0.05 and a posterior probability of H4 (PPH4) > 0.60. The heterogeneity in the dependent instruments (HEIDI) test was applied to assess heterogeneity among instrumental variables. MDD, major depressive disorder; BD, bipolar disorder; SCZ, schizophrenia.

The SMR results assessing associations between OSGs expression and the three psychiatric disorders are shown in Figure 3 and Supplementary File: Table S4. After multiple testing correction, significant associations were found for 19 OSGs with MDD, among which 2 OSGs demonstrated colocalization support. Specifically, a 1 SD increase in the expression of *CPT1A* (OR = 0.951, 95% CI: 0.930–0.974; PPH4 = 0.83) and *SEPN1* (OR = 0.906, 95% CI: 0.851–0.964; PPH4 = 0.73) was associated with a 4.9% and 9.4% reduction in MDD risk, respectively. For BD, 16 OSGs showed significant associations, with 3 OSGs supported by colocalization evidence. Increased expression of *GGT1* (OR = 0.871, 95% CI: 0.811–0.935, PPH4 = 0.81) and *NDUFS2* (OR = 0.86, 95% CI: 0.801–0.924, PPH4 = 0.91) was linked to a reduced BD risk, whereas elevated *CHUK* expression corresponded to an increased risk (OR = 1.295, 95% CI: 1.124–1.492, PPH4 = 0.81). In SCZ, although 24 OSGs showed statistically significant associations in the SMR analysis, no genes supported by colocalization evidence, suggesting that the observed associations may not reflect shared causal variants.

### 3.3. Blood OSGs-Encoded Protein Abundance Level and Major Psychiatric Disorders

**Figure 4:**
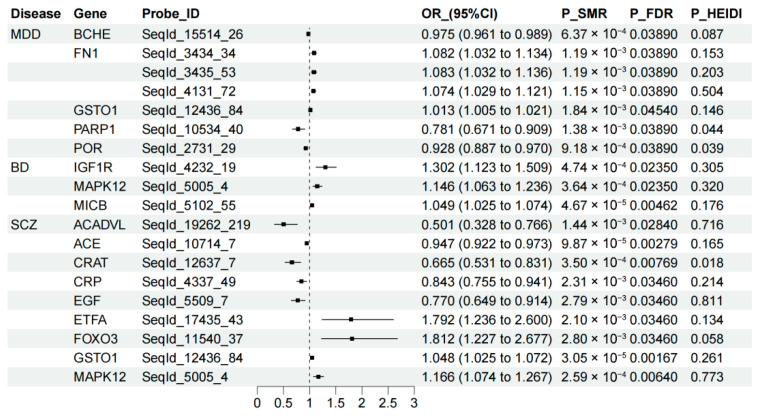
Association of oxidative stress-related gene-encoded protein abundance with psychiatric disorders. The figure presents findings from summary-data-based Mendelian randomization (SMR) analysis, showing associations that met the threshold of false discovery rate (FDR)-adjusted *p* < 0.05 and a posterior probability of H4 (PPH4) > 0.60. The heterogeneity in the dependent instruments (HEIDI) test was applied to assess heterogeneity among instrumental variables. MDD, major depressive disorder; BD, bipolar disorder; SCZ, schizophrenia.

The SMR results examining associations between OSGs-encoded protein abundance levels and psychiatric disorders are summarized in Figure 4 and Supplementary File: Table S5. After multiple testing correction, seven OSGs showed significant associations with MDD at the protein abundance level, but none were supported by the colocalization evidence, suggesting potential linkage effects rather than shared causal variants. For BD, several OSGs demonstrated significant associations, with one gene supported by colocalization. Specifically, a 1-SD increase in the genetically predicted protein abundance of *IGF1R* was associated with a 30.2% higher risk of BD (OR = 1.302, 95% CI: 1.123–1.509, PPH4 = 0.72). In SCZ, nine OSGs exhibited significant associations, with one gene showing colocalization support. Elevated genetically predicted *FOXO3* protein abundance was significantly associated with an increased SCZ risk (OR = 1.812, 95% CI: 1.227–2.677; PPH4 = 0.77).

### 3.4. Brain Tissue Validation of Causal Associations Across Different Levels

The brain-specific SMR results examining associations between DNA methylation of OSGs and these three psychiatric disorders are presented in Supplementary File: Table S3. Overall, the majority of significant loci across genes were consistent with those identified in blood tissue. After multiple testing correction, we identified 89 significant CpG loci near 54 OSGs associated with MDD, among which 31 loci across 22 genes were supported by colocalization evidence. For SCZ, 174 significant CpG loci near 95 OSGs were identified, with 56 loci across 38 genes showing colocalization support. In BD, 66 significant CpG loci near 33 OSGs were detected, and 39 loci across 18 genes demonstrated colocalization evidence.

The brain-specific SMR results assessing associations between OSGs expression and these three psychiatric disorders are shown in Supplementary File: Table S4. After multiple testing correction, significant associations were identified for 4 OSGs related to MDD, although none were supported by colocalization evidence. For BD, 6 OSGs showed significant associations at the gene expression level, with 2 genes showing colocalization support. However, no overlapping genes were observed between brain- and blood-based analyses. In brain tissue, elevated expression of *PRKCA* (OR = 1.121, 95% CI: 1.053–1.194; PPH4 = 0.89) and *STK4* (OR = 1.180, 95% CI: 1.113–1.252; PPH4 = 0.97) was significantly associated with increased BD risk. For SCZ, 28 OSGs showed significant associations, with 6 genes (*ACADVL*, *ACE*, *C12orf65*, *CD40*, *GCH1*, and *STK4*) showing colocalization support. Among these, findings for *ACADVL* (OR = 0.932, 95% CI: 0.897–0.968; PPH4 = 0.73), *ACE* (OR = 0.927, 95% CI: 0.897–0.957; PPH4 = 0.99), and *GCH1* (OR = 0.926, 95% CI: 0.891–0.962; PPH4 = 0.76) were consistent with blood-based results, where higher gene expression was associated with reduced SCZ risk.

The brain-specific SMR results examining associations between OSGs-encoded protein abundance and these three psychiatric disorders are summarized in Supplementary File: Table S5. After multiple testing correction, we identified 1, 7, and 9 OSGs significantly associated with MDD, BD, and SCZ, respectively. Among these, no genes for MDD, two genes for BD, and two genes for SCZ were supported by colocalization evidence. For BD, both *MAPK3* (OR = 1.216, 95% CI: 1.122–1.317; PPH4 = 0.95) and *PRKCB* (OR = 1.108, 95% CI: 1.051–1.169; PPH4 = 0.91) were identified as significant in brain tissue, but were not detected in blood. In contrast, findings for schizophrenia were highly consistent with across tissues: increased *ACADVL* (OR = 0.881, 95% CI: 0.817–0.950; PPH4 = 0.80) and *ACE* (OR = 0.896, 95% CI: 0.846–0.950; PPH4 = 0.98) protein abundance in the brain was significantly associated with a reduced risk of SCZ, mirroring the direction and magnitude of effects observed in blood.

### 3.5. Multi-Omics Evidence Integration at Tissue Level

As shown in Table 1, integration of SMR results across multiple gene regulation levels in blood tissue prioritized *IGF1R* as a Tier 2 candidate gene for BD, although this association was not validated in brain tissue. In contrast, *ACE* and *ACADVL* were prioritized as Tier 3 candidate genes for SCZ based on blood multi-omics data, and both two genes were validated in brain tissue with stronger supporting evidence. Notably, these prioritizations remained unchanged in sensitivity analyses, indicating the robustness of the findings (Supplementary File: Tables S7–S9).

### 3.6. Identifying Immune and Brain Cell-Specific Effects of Identified OSGs Expression on Psychiatric Disorders

It is worth noting that a 1-SD increase in genetically regulated *ENDOG* expression was significantly associated with increased SCZ risk across multiple brain and immune cell types. Specifically, higher *ENDOG* expression was linked to 8.9%, 9.9%, 9.2%, and 13.1% increase in the SCZ risk in astrocytes (OR = 1.089, 95% CI: 1.024–1.159), CD4+ naive T cells (OR = 1.099, 95% CI: 1.041–1.161), CD8+ effector T cells (OR = 1.092, 95% CI: 1.026–1.163), and natural killer (NK) cells (OR = 1.131, 95% CI: 1.049–1.22), respectively. Although none of these associations met the pre-defined colocalization threshold (PPH4 ≥ 0.7), the observed convergence across multiple cell types provides supportive evidence for *ENDOG*’s potential role in SCZ at the cellular level, rather than establishing definitive causality. This multi-cellular convergence pattern aligns closely with the integrated multi-omics findings (Supplementary File: Table S6). These findings were robust to sensitivity analyses (Supplementary File: Table S10).

## 4. Discussion

This study conducts a systematic, multi-omics investigation of the potential links between OSGs and three major psychiatric disorders (MDD, BD, and SCZ). By integrating summary-level data from blood and brain tissues with single-cell eQTL resources, we implemented an analytical framework to evaluate and prioritize candidate genes within oxidative stress pathways. Our analyses revealed a clear spectrum of evidence strength across disorders. The most consistent and robust findings were observed for SCZ, where ACE and ACADVL were prioritized as high-priority candidate genes, with genetically predicted associations supported across tissues and, notably, by strong colocalization evidence at the brain protein level. For BD, IGF1R showed association primarily in blood-based analyses, a finding not replicated in brain tissue, which may indicate tissue-specific mechanisms or reflect current limitations in brain pQTL statistical power. For MDD, the convergence of evidence under our stated colocalization criteria was weaker, necessitating a more cautious interpretation and highlighting this disorder as a key target for future studies with enhanced power or refined phenotyping. In brain-tissue analyses, ENDOG was implicated as a candidate gene for SCZ risk, and single-cell MR analyses suggested a potential role in specific immune and glial cell types, although this cell-type-specific evidence remains preliminary and requires further validation. Collectively, these findings identify a set of prioritized candidate genes and pathways linking oxidative stress to psychiatric disorders, offering a resource and rationale for future functional studies to explore their mechanistic roles and potential therapeutic relevance.

While Tier 1 genes represent the highest-confidence candidates supported by evidence across all three molecular layers, genes in Tiers 2 and 3 also hold significant biological and translational interest. Their association patterns, converging across either two layers or observed at the critical protein level and one layer alone, may reflect several meaningful scenarios. These include technical limitations in current QTL datasets (e.g., power or tissue specificity), the biological reality of disease heterogeneity, or cell-type-specific regulatory mechanisms. For instance, a protein-level association without corresponding cis-mQTL or eQTL support could point to post-transcriptional regulation or highlight that gene as a potential marker for a specific disease subtype. Therefore, these genes should not be disregarded but considered as a valuable pool for future hypothesis-driven research. Their validation in independent cohorts and functional studies, particularly in the context of disentangling disease subtypes or differential treatment responses, will be a crucial next step to unlock their full therapeutic potential.

The cross-tissue consistency for *ACE* and *ACADVL* in both brain and blood tissues may imply their translational relevance. From a biomarker perspective, our findings suggest that their expression or protein levels in blood, which is an accessible peripheral tissue, may serve as measurable proxies reflecting shared pathogenic processes with the brain, potentially aiding in risk stratification or subtyping. More importantly, these genes may have great potential to translate as therapeutic targets. Consistent with previous findings that reduced ACE expression in blood and prefrontal cortex is associated with an increased risk of SCZ [32], our study demonstrated that elevated *ACE* blood DNA methylation, gene expression, and protein abundance were significantly associated with decreased SCZ risk. These associations were replicated in both blood and brain tissue, underscoring the robustness of the finding. Notably, for several genes (e.g., *ACADVL*), we observed that increased methylation at different CpG sites was associated with opposite directions of effect on disease risk. This phenomenon highlights the context-dependent nature of DNA methylation regulation. Methylation can exert activating or repressive effects on gene expression based on the specific genomic features it influences. A CpG site located within a transcriptional enhancer may, when methylated, silence that enhancer and reduce gene expression [33]. Conversely, methylation within a repressive element or a region governing alternative promoter usage might lead to increased expression of a specific, perhaps functional, transcript isoform [34]. Therefore, the opposing risk associations for different CpG sites may reflect its complex regulatory architecture, where individual sites modulate distinct aspects of its transcriptional output. Future studies integrating allele-specific expression, chromatin conformation, and fine-mapping of regulatory elements are needed to dissect these mechanisms definitively. *ACE* encodes a key enzyme in the renin–angiotensin system (RAS), which converts angiotensin I into angiotensin II. Beyond its cardiovascular functions, *ACE* may exert neuroprotective effects by modulating neuronal signaling and oxidative balance through RAS-dependent mechanisms [32,35]. Our genetic evidence, aligning with prior observational studies, raises the hypothesis that modulating *ACE* activity could influence SCZ risk through neuroprotective mechanisms, warranting investigation in pre-clinical models. Although the methylation-SCZ associations for *ACADVL* varied across CpG sites, we consistently observed that higher *ACADVL* expression and protein abundance were associated with reduced SCZ risk across both blood and brain tissue datasets. *ACADVL* encodes a mitochondrial enzyme essential for very long-chain fatty acids (VLCFA) β-oxidation, a process critical for cellular energy metabolism [36]. Proper *ACADVL* function maintains fatty acid homeostasis, while its dysfunction can lead to *VLCFA* accumulation, lipo-toxicity, and neuronal impairment [36,37], mechanisms plausibly linking mitochondrial metabolism to psychiatric vulnerability. Pharmacological strategies aimed at enhancing mitochondrial function or correcting VLCFA metabolism could be explored as novel therapeutic avenues. Future research should prioritize functional validation of these genes in neural models and investigate whether their products or pathways can be modulated for therapeutic benefit.

Our findings also supported the regulatory role of *IGF1R* in BD pathogenesis, although the association was not validated in brain tissue. *IGF1R* signaling modulates autophagy and oxidative phosphorylation and is essential for neuronal survival, synaptic plasticity, and cognitive function [38,39]. Previous research has shown that rare variants in *GIGYF1*, a gene that enhances insulin and *IGF1R* signaling, are associated with metabolic abnormalities, a frequent comorbidity in BD [40]. This discrepancy between blood and brain findings may arise from several, non-mutually exclusive factors. It could reflect a genuine tissue-specific mechanism, wherein IGF1R influences BD risk primarily through peripheral (e.g., immunometabolic) pathways, which is consistent with the well-established link between BD and systemic metabolic dysregulation [35]. Alternatively, it may be partly attributable to the more limited statistical power of current brain pQTL datasets compared to blood resources, increasing the risk of false negatives for genuine brain-specific associations. This underscores the importance of interpreting causal inferences within their specific tissue context and the need for larger brain molecular QTL maps. Taken together, these results suggest that *ACE*, *ACADVL*, and *IGF1R* represent causal and biologically plausible mediators linking oxidative stress to psychiatric disorders. Future studies employing experimental validation and targeted functional assays are warranted to determine whether modulating these OSGs or their molecular pathways could serve as promising therapeutic strategies for the prevention and management of psychiatric disorders.

In the brain tissue-based analysis, *ENDOG* gene emerged as significant association with SCZ across multiple gene regulation levels, suggesting a potential causal role in disease pathogenesis. Although large-scale genomic studies have not directly implicated *ENDOG* in SCZ, converging biological evidence supports its functional relevance. Endonuclease G (EndoG), encoded by *ENDOG* gene, is a mitochondrial nuclease that plays a critical role in mitochondrial DNA (mtDNA) replication and recombination. Experimental studies have shown that reduced expression of *ENDOG* leads to mitochondrial ROS accumulation, impaired cell proliferation, and disrupted mitochondrial homeostasis [41,42,43]. Given that mitochondrial dysfunction is increasingly recognized as a core pathogenic process in psychiatric disorders [44,45], dysregulation of *ENDOG* at the epigenetic, transcriptional, or proteomic level may contribute to SCZ vulnerability by compromising mitochondrial integrity and energy metabolism. While these findings should be interpreted cautiously regarding brain-specific mechanisms, in an exploratory analysis, our single-cell SMR findings seem to lend supportive evidence by showing consistent associations between *ENDOG* expression and elevated SCZ risk across multiple cell types, including astrocytes, CD4^+^ naïve T cells, CD8^+^ effector T cells, and NK cells. In CD4+ and CD8+ T lymphocytes, *ENDOG* has been shown to suppress telomerase activity by via regulation of *hTERT* alternative splicing, leading to telomere shortening and cellular senescence [46]. In SCZ, this mechanism may synergize with immune regulation abnormalities such as Klotho protein downregulation [47], accelerating premature cellular aging, a process frequently observed in SCZ patients. Clinical interventions like Omega-3 fatty acid supplementation have shown their potential to modulate telomerase and oxidative stress pathways, offering a translational avenue for targeting these processes in SCZ [48]. Moreover, *ENDOG* regulates immune cell proliferation and function by modulating ROS production, and excessive ROS can amplify inflammatory cascades characterized by elevated tumor necrosis factor (TNF) and interleukin-6 (IL-6) expression [49]. These peripheral inflammatory responses may, in turn, affect cerebral endothelial cells and microglial activation, contributing to neuroinflammation and neuropathological changes implicated in SCZ development [50,51]. Beyond its roles in autophagy regulation, oxidative stress management, and DNA damage response, *ENDOG* may therefore serve as a molecular nexus linking mitochondrial dysfunction, immune dysregulation, and cellular senescence in SCZ. It is necessary to acknowledge that due to the absence of strong colocalization, the single-cell SMR analyses implicating *ENDOG* should be interpreted as supportive evidence. Future mechanistic and functional studies, particularly those employing cell-type-resolved transcriptomic and proteomic profiling, are warranted to establish definitive causality and clearly clarify *ENDOG*’s contribution to disease heterogeneity and therapeutic response.

The major strength of this study lies in its use of a comprehensive multi-omics framework to systematically investigate causal associations between OSGs and the three major psychiatric disorders across multiple gene regulation levels. By integrating blood- and brain-specific multi-omics data with single-cell eQTL datasets, this work provides a multiscale perspective that enhances understanding of the regulatory architecture underlying psychiatric risk and offers insights into precision therapeutic targeting. Nevertheless, several limitations of this study should be acknowledged. First, the statistical power for discovering brain-specific associations was constrained by the comparatively smaller sample sizes of available brain QTL datasets relative to blood-based resources. Second, the predominant use of GWAS and QTL data from European-ancestry cohorts limits the generalizability of our findings to other populations, highlighting a critical need for more diverse molecular resources in future research. Third, our analytical focus was restricted to a pre-defined set of OSGs from the GeneCards database. While this provided a structured, hypothesis-driven framework, it may introduce selection bias by excluding relevant genes not cataloged under this specific term. Fourth, our requirement for protein-level significance, while strengthening biological interpretability, represents a conservative trade-off that may have further limited discovery, especially given the power constraints of brain pQTL datasets mentioned above. Fifth, to minimize violations of the SMR pleiotropy assumption, our analysis was confined to cis-acting QTLs, potentially missing long-range regulatory effects mediated by trans-QTLs [16]. Sixth, our single-cell MR findings must be interpreted in the context of current methodological constraints. The statistical power and cellular resolution of pioneering single-cell eQTL resources (e.g., DICE and OneK1K), while groundbreaking, remain limited by cohort size and may not capture all disease-relevant cell states or populations, which could affect the comprehensiveness and detection of cell-type-specific associations. Seventh, the biological interpretation of discordant findings between blood and brain tissues (e.g., for IGF1R) remains challenging, as it could reflect true tissue-specific pathophysiology, differences in statistical power, or a combination thereof. Finally, while our multi-omics integration criteria prioritized high-confidence candidate genes, definitive establishment of their biological relevance and causal roles requires direct experimental validation through in vitro and in vivo functional studies.

## 5. Conclusions

In conclusion, this study systematically explored the causal associations between OSGs and the three major psychiatric disorders, MDD, BD, and SCZ, across multiple gene regulation levels. By integrating blood- and brain-specific multi-omics data with single-cell eQTL datasets, we identified *ACE* and *ACADVL* as key causal genes contributing to SCZ pathogenesis, and *IGF1R* as a candidate gene associated with BD risk. In brain tissues, the role of *ACE* and *ACADVL* were further validated, and *ENDOG* was identified as a gene significantly linked to SCZ susceptibility. At the single-cell level, *ENDOG* expression was positively associated with SCZ risk across multiple cell types, including astrocytes, CD4+ naive T cells, CD8+ effector T cells, and NK cells. Collectively, these findings advance understanding of the oxidative stress–immune–mitochondrial axis in psychiatric disorders and provide a molecular foundation for the development of targeted therapeutic interventions. Future studies could build upon our prioritized gene list by employing advanced causal inference frameworks, such as multivariable Mendelian randomization, to disentangle the independent and potentially mediated contributions of epigenetic, transcriptomic, and proteomic changes within specific biological pathways to psychiatric risk.

## Figures and Tables

**Table 1:** Multi-omics integration of oxidative stress-related genes across DNA methylation, expression, and protein levels in psychiatric disorders.

xQTL Source	Outcome	Gene	Level	mQTL				eQTL			pQTL			
				Probe	OR (95%CI)	P_SMR	P_FDR	OR (95%CI)	P_SMR	P_FDR	OR (95%CI)	P_SMR	P_FDR	PPH4
Blood	BD	*IGF1R*	Tier 2	cg15903819	1.10 (1.04, 1.17)	1.05 × 10^−3^	0.030	1.02 (0.93, 1.11)	7.22 × 10^−1^	0.944	1.30 (1.12, 1.51)	4.74 × 10^−4^	0.023	0.72
				cg20891771	1.05 (1.02, 1.09)	1.94 × 10^−3^	0.040							
	SCZ	*ACADVL*	Tier 3	cg19466160	0.87 (0.81, 0.95)	8.67 × 10^−4^	0.020	0.90 (0.85, 0.95)	1.95 × 10^−4^	0.005	0.50 (0.33, 0.77)	1.44 × 10^−3^	0.028	0.70
				cg16564940	0.87 (0.79, 0.95)	2.65 × 10^−3^	0.041							
				cg13768953	1.05 (1.03, 1.08)	2.31 × 10^−4^	0.006							
				cg03613822	1.04 (1.02, 1.06)	2.01 × 10^−4^	0.005							
		*ACE*	Tier 3	cg21657705	0.92 (0.88, 0.96)	2.32 × 10^−4^	0.010	0.57 (0.44, 0.75)	4.41 × 10^−5^	0.001	0.95 (0.92, 0.97)	9.87 × 10^−5^	0.003	0.66
				cg04199256	0.84 (0.74, 0.94)	2.80 × 10^−3^	0.043							
				cg01719825	0.85 (0.77, 0.93)	3.27 × 10^−4^	0.008							
Brain	SCZ	*ACADVL*	Tier 1	cg12228229	1.13 (1.05, 1.21)	1.14 × 10^−3^	0.020	0.93 (0.90, 0.97)	2.71 × 10^−4^	0.007	0.88 (0.82, 0.95)	9.92 × 10^−4^	0.018	0.80
		*ACE*	Tier 2	/	/	/	/	0.93 (0.90, 0.96)	4.17 × 10^−6^	0.000	0.90 (0.85, 0.95)	2.18 × 10^−4^	0.008	0.98
		*ENDOG*	Tier 3	cg04621255	0.91 (0.87, 0.96)	4.51 × 10^−4^	0.010	1.05 (1.02, 1.08)	1.65 × 10^−4^	0.005	1.07 (1.03, 1.11)	3.99 × 10^−4^	0.011	0.53
				cg13630871	0.96 (0.93, 0.98)	2.82 × 10^−3^	0.039							

Note: We integrate evidence from three molecular levels: DNA methylation (methylation quantitative trait loci, mQTL), gene expression (expression quantitative trait loci, eQTL), and protein abundance (protein quantitative trait loci, pQTL). Associations were tested using summary-data-based Mendelian randomization (SMR), with significance assessed after false discovery rate (FDR) adjustment. P_SMR denotes the raw *p*-value from the SMR test, and P_FDR represents the FDR-adjusted *p*-value. MDD, major depressive disorder; BD, bipolar disorder; SCZ, schizophrenia.

## Data Availability

All the data used in the present study were publicly available on websites. The GWAS summary statistics for psychiatric disorders can be downloaded from the PGC (https://pgc.unc.edu (accessed on 5 February 2026)). The GWAS summary statistics on expression were provided by Võsa U. et al. and de Klein N. et al. from (https://www.eqtlgen.org/cis-eqtls.html (accessed on 5 February 2026)) and (https://yanglab.westlake.edu.cn (accessed on 5 February 2026)). The GWAS summary statistics on methylation were based on publicly available summarized data by McRae AF. et al. and Ng B. et al. from (https://yanglab.westlake.edu.cn (accessed on 5 February 2026)) and (http://mostafavilab.stat.ubc.ca/xQTLServe (accessed on 5 February 2026)). The GWAS summary statistics on protein can be obtained by Ferkingstad E. et al. and Wingo AP. et al. from (https://www.decode.com/summarydata/ (accessed on 5 February 2026)) and (https://doi.org/10.7303/syn51150434).

## Supplementary Materials

The following supporting information can be downloaded at https://www.mdpi.com/article/10.3390/antiox15020233/s1; Table S1: 817 OS-related genes with a relevance score ≥ 7 obtained from GeneCards. Table S2: Information of included studies and consortia. Table S3: Summary-data-based mendelian randomization and colocalization results of the association between oxidative stress-related DNA methylations and psychiatry disorders (HEIDI test *p* > 0.01). Table S4: Summary-data-based mendelian randomization and colocalization results of the association between oxidative stress-related gene expression and psychiatry disorders (HEIDI test *p* > 0.01). Table S5: Summary-data-based mendelian randomization and colocalization results of the association between oxidative stress-related gene-encoded protein abundance level and psychiatry disorders (HEIDI test *p* > 0.01). Table S6: Summary-data-based mendelian randomization and colocalization results of the association between oxidative stress-related gene expression and single-cell (HEIDI test *p* > 0.01). Table S7: Summary-data-based mendelian randomization and colocalization results of the association between oxidative stress-related DNA methylations and psychiatry disorders (HEIDI test *p* > 0.05). Table S8: Summary-data-based mendelian randomization and colocalization results of the association between oxidative stress-related gene expression and psychiatry disorders (HEIDI test *p* > 0.05). Table S9: Summary-data-based mendelian randomization and colocalization results of the association between oxidative stress-related gene-encoded protein abundance level and psychiatry disorders (HEIDI test *p* > 0.05). Table S10: Summary-data-based mendelian randomization and colocalization results of the association between oxidative stress-related gene expression and single-cell (HEIDI test *p* > 0.05).

## Abbreviations

The following abbreviations are used in this manuscript:

MDD	Major Depressive Disorder
BD	Bipolar Disorder
SCZ	Schizophrenia
OS	Oxidative Stress
ROS	Reactive Oxygen Species
HCs	Healthy Controls
GWASs	Genome-Wide Association Studies
QTL	Quantitative Trait Loci
MR	Mendelian Randomization
SMR	Summary Data-Based MR
mQTLs	DNA Methylation QTLs
eQTLs	Expression QTLs
pQTLs	Protein QTLs
OSGs	OS-Related Genes
SNPs	Single Nucleotide Polymorphisms
scRNA-seq	single-cell RNA sequencing
PBMCs	Peripheral Blood Mononuclear Cells
HEIDI	Heterogeneity in Dependent Instruments
LD	Linkage Disequilibrium
PPH4	Posterior Probability for Hypothesis 4
SD	Standard Deviation
RAS	Renin–Angiotensin System
VLCFA	Very Long-Chain Fatty Acids
EndoG	Endonuclease G
mtDNA	Mitochondrial DNA
TNF	Tumor Necrosis Factor
IL-6	Interleukin-6
